# Physical Fitness—Not Physical Activity Levels—Influence Quality of Life in Anorexia Nervosa

**DOI:** 10.3390/ijerph19052678

**Published:** 2022-02-25

**Authors:** Alexa Agne, Hugo Olmedillas, Margarita Pérez Ruiz, Miguel del Valle Soto, Maria Fernandez-del-Valle

**Affiliations:** 1 Department of Applied Health, Southern Illinois University Edwardsville, Edwardsville, IL 62026, USA; aleagne@siue.edu; 2 Department of Functional Biology, Campus del Crito B, University of Oviedo, 33006 Oviedo, Spain; olmedillashugo@uniovi.es; 3 Health Research Institute of the Principality of Asturias (ISPA), 33011 Oviedo, Spain; miva@uniovi.es; 4 Grupo de Investigación de Investigación en Nutrición, Ejercicio y Estilo de Vida Saludable (ImFINE), Departamento de Salud y Rendimiento, Facultad de Ciencias de la Actividad Física y del Deporte–Instituto Nacional de Educación Física (INEF), Universidad Politécnica de Madrid, 28040 Madrid, Spain; margarita.perez@upm.es; 5 Department of Morphology and Cellular Biology, Anatomy, Campus del Crito B, University of Oviedo, 33006 Oviedo, Spain

**Keywords:** anorexia nervosa, muscular fitness, quality of life, physical activity, physical fitness

## Abstract

Background: Incorporating physical activity (PA) has been a challenge for health care professionals working with anorexia nervosa (AN) patients. This has contributed to partial physical fitness (PFit) recovery that persists after weight restoration. Objective: This cross-sectional study aimed to examine the relationships between PA, sedentary behaviors, PFit, and quality of life (QoL) in a group of adolescents after hospitalization. Methods: QoL, PA, and sedentary behaviors were measured using the Health-Questionnaire Short-Form 36 (SF-36) and accelerometers, while PFit was assessed through cardiorespiratory fitness, body composition (anthropometry), and strength (six repetition maximum) tests in a total of 63 patients. Results: Light-PA (LPA), moderate-PA (MPA), moderate-to-vigorous-PA (MVPA), and relative sedentary time (%ST) did not meet the recommendations (*p* < 0.001). Only 22% of the patients met MVPA criteria, and ~82% exceeded %ST. SF-36 scores were lower than normative values except the physical component scale. Absolute cardiorespiratory fitness was reduced (*p* < 0.001) in 84% of the patients, and was positively associated to body weight, body mass index (BMI), circumferences, and muscle areas. Additional positive significant relationships were found between QoL, muscular strength, and body composition, and negative associations between vigorous-to-very vigorous PA and BMI, skinfolds, and percent body fat. Regression analyses revealed lower body strength as an explanatory factor for improved QoL (*OR* 1.03, 95%CI 1.00–1.07). Conclusions: PFit and QoL scores are poor after hospitalization. LPA, MPA, and MVPA do not meet recommendations. PFit management—with emphasis on improving muscular fitness—may be a valuable strategy for QoL improvement in AN after hospitalization.

## 1. Introduction

Anorexia nervosa (AN) is an eating disorder characterized by a fear of weight gain and an extreme drive for thinness [1]. Physical activity (PA) has been a controversial topic in AN. Around 31% of patients engage in unhealthy PA (uPA) in early stages of the disease as a weight loss strategy, which increases up to 80% prior to hospitalization. It is often the first presenting and last remaining symptom in this population [2,3,4], and engagement in uPA after treatment is a relapse predicting eating disorder symptom [5]. For this reason and to reduce the metabolic demand, bed rest and exercise restriction has historically been the elected strategy in AN patients [6]. Bed rest has shown to negatively impact bone health during hospitalization [7], while low-mechanical stimulus (exercise) is able to prevent bone turnover decline in AN [8]. More importantly, these practices reduce treatment acceptability and produce negative perceptions which harm engagement with treatment [9,10]. Overall, patients in critical care experience an increase of pro-inflammatory cytokines and reactive oxygen species, leading to muscle proteolysis and promoting muscle mass and strength loss [11]. More current knowledge suggests that any level of PA viable in patients with AN should be preserved during all stages of treatment [12,13]. More specifically, nutritionally supported strength-based exercise of moderate-to-high intensity has proven useful for enhancing mental health and physical fitness (PFit) in AN patients [5,14,15,16,17], without negatively impacting feelings about food or weight recovery [18,19,20,21].

Incorporating exercise has been a challenge for health care teams due to a lack of guidelines and insufficient knowledge on safe and effective exercise in AN [13], therefore, resulting in decreased PA levels [12,22]. This has contributed to partial PFit recovery, even after weight or body mass index (BMI) restoration [6]. PFit, also referred to as “health status”, is affected by physical activity behaviors. In AN, PFit health-related components are negatively affected with reduced cardiorespiratory (CR) function, muscular fitness, decreased functional mobility—reduced performance in activities of daily living—and altered body composition (i.e., reduced skinfold thicknesses and circumferences at the extremities, low muscle mass, and bone mineral density at different stages of the treatment) that persists even after weight and BMI recovery and impacts negatively quality of life (QoL) [2,6,15,23].

Subjective tools such as self-reported questionnaires or interviews have proven to either over-estimate or under-estimate PA levels in AN patients and healthy populations [24,25]. In a study conducted during acute treatment—using both subjective (questionnaires) and objective (accelerometers) tools to measure PA—PA levels from questionnaires were significantly below values obtained from accelerometers [25]. The assessment of PA requires objective tools; however, there are no standardized values for healthy PA levels in AN patients. In addition, uPA has not been fully defined until recently, uncovering that the nature of the behavior (qualitative dimension: motives, compulsivity, etc.) and not intensity or duration alone (quantitative dimension) might be reflective of uPA [4]. 

Sedentary time (ST) has emerged as an independent factor affecting PFit, all-cause mortality, and cardiovascular disease risk in general population regardless of PA levels [26]. Wear time is the minimum amount of time an individual has to wear an accelerometer to be considered for examination and allows for reliable quantification of PA and ST. Adolescents with ST relative to wear time (%ST) > 69% (75%, males) display significantly lower PFit irrespective of the PA levels [27]. Therefore, assessment and management of both ST and PA is of great importance in the development of strategies for health improvement in AN.

To the best of our knowledge, there are no studies that have investigated PA and sedentary behavior after acute care, nor the relationship with PFit levels and perceived QoL in patients with AN. Therefore, the purpose of our study was to examine PA and sedentary behaviors and the relationship with PFit and QoL in a group of adolescents with AN after acute treatment.

## 2. Materials and Methods

**Figure 1:**
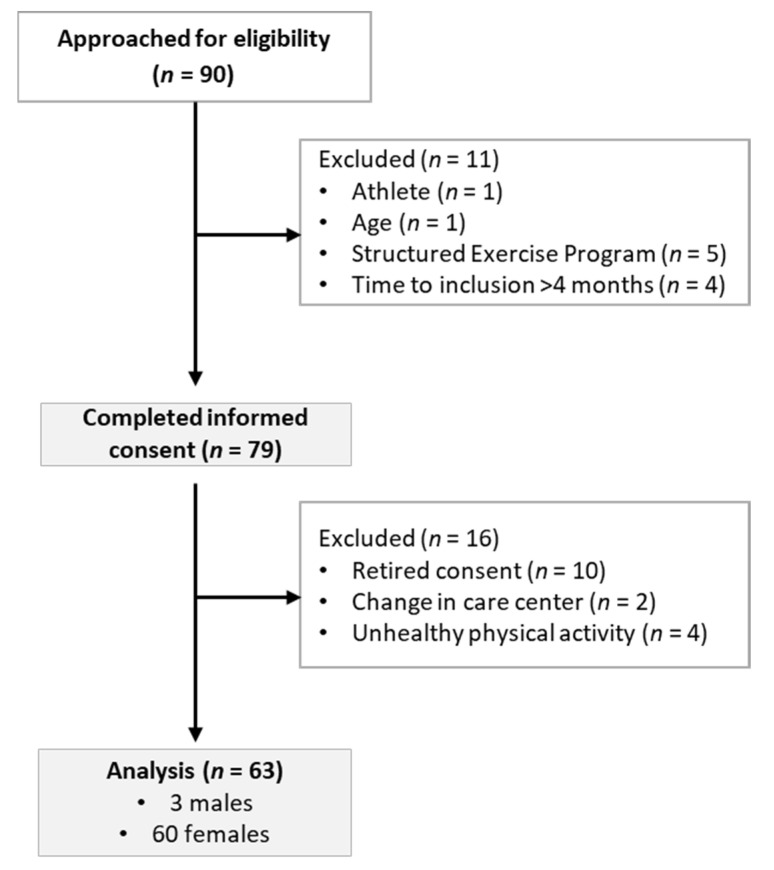
Flow diagram of study.

This is an observational study with a cross-sectional design that followed STROBE guidelines for observational studies [28]. Participants were recruited through convenience sampling. Inclusion criteria consisted of: restricting type of AN [29]; age 12–18 years old, BMI > 14.0 (kg/m^2^) [30], participation in regular treatment (partial outpatient regime), and enrolled within four months after admission to acute treatment. Exclusion criteria consisted of: participating in structured exercise programs, engaging in uPA, being an athlete [31], and having contraindications to perform exercise. The protocol was approved by the Ethics Committee of the hospital (ID: R-0034/08), and parental consent was obtained. The study was carried out in accordance with The Declaration of Helsinki for Human Research. A total of 90 patients were approached for eligibility, from which 11 were excluded: one was an athlete, one did not meet the age requirement, five were participating in structured exercise, and the time from admission to inclusion was >4 months in four patients. A total of 79 were enrolled in the study, and 16 were excluded due to the following reasons: ten participants withdrew consent due to concerns related to accelerometry assessment, two participants changed care center, and four participants engaged in uPA. Therefore, a sample of 63 patients (3 males) was analyzed (see Figure 1).

**Figure 2:**
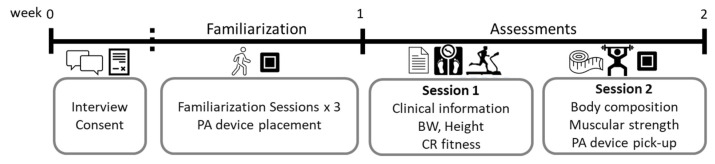
Chronogram of the study. PA—physical activity; BW—body weight; CR—cardiorespiratory.

All assessments were completed in two sessions under similar conditions and time (20–24 °C, 45–55% relative humidity, 9:00 a.m.–11:00 p.m.). Participants consumed their usual breakfast [fruit juice (~200 cc) and a bowl of cereal (~45 g) with milk (~200 cc)] three hours before the assessments. The assessments were conducted by the same researcher, except for the cardiorespiratory fitness test which was carried out and monitored by a physician. Figure 2 depicts the organization of the familiarization and all the assessments performed.

### 2.1. Physical Activity Assessment

PA levels were quantified using accelerometers Actigraph (Model 7164 MTI Health Service, Fort Walton, FL, USA, EEUU). Patients were instructed to wear the accelerometer for 10 days, and valid days were considered those that registered a minimum of 8 h. A total of three working days (working/school days) and two weekend holidays (non-working/school days) were selected [32]. ST and four levels of PA [light PA (LPA), moderate PA (MPA), vigorous PA (VPA), and very vigorous PA (VPA)] were calculated according to the cut-off points for children under 15 years [33] and for those over 15 years of age [34].

### 2.2. Physical Fitness Assessments

In order to avoid injuries and to prepare the musculoskeletal structures for the assessments, a familiarization period including three 50-minute sessions was completed by all participants prior to the assessment week. The sessions included a warmup, cool down, and main session (i.e., 2–3 sets of 5–8 repetitions of the exercises (unloaded) used to assess muscular strength, 2–3 sets of the functional mobility tests, and one set for the treadmill ergometer). All of the assessment sessions were preceded by a warmup and followed by a cool down.

#### 2.2.1. Cardiorespiratory Fitness

A peak oxygen consumption (VO_2_peak) test on a treadmill (Technogym Run Race 1400HC; Gambettola, Italy) was performed—in conjunction with an ECG (BTL-08MT Plus ECG). The treadmill started at 3.0 km/h and an incline of 5.0%, and both speed and incline were increased by 0.3 km/h and 0.5% every 30 s. The test was terminated when participants could not maintain the workload or until volitional fatigue [15]. 

#### 2.2.2. Muscular Strength and Functional Mobility

A six-repetition maximum test (6RM) was performed in the upper body [bench press (6RM-BP) and seated lateral row (6RM-LR)] and lower body [leg press (6RM-LP)] following a standardized protocol using pediatric resistance weight machines (Strive Inc., Philadelphia, PA, USA). To measure functional mobility, we used the Timed Up and Go 3 m (TUG-3m) and 10 m (TUG-10m), and the Timed Up and Down Stairs (TUDS). Details on these tests are provided elsewhere [15]. 

#### 2.2.3. Body Composition

Body weight (BW), height, biceps, triceps, subscapular, suprailiac, abdominal, thigh, and medial calf skinfolds (mm), and mid-thigh, arm, and calf circumferences (cm) were measured by a certified Level 3 technician following the standardized procedures endorsed by the International Society for de Advancement of Kinanthropometry [35]. The following variables were calculated: BMI (kg/m^2^), %BF (specific for patients with AN) [36]; skeletal muscle mass (SMM) [37], arm, thigh and calf muscle cross-sectional areas (CSA) [38], and sum of six skinfolds (Sum6-SK): biceps, triceps, subscapular, suprailiac, mid-thigh, and medial calf. 

#### 2.2.4. Diet Management

The nutritional management of the patients (dietary intake) varied from patient to patient and was handled by the medical team. The dietary intake was recorded as kcals/day during the assessment period.

### 2.3. Quality of Life Assessment

QoL was evaluated using the Health Questionnaire Short-Form 36 (SF-36). The SF-36 has eight domains: physical functioning (PF), role limitations due to physical health (RP), bodily pain (BP), general health (GH), vitality (VT), social functioning (SF), role limitations due to emotional problems (RE), and mental health (MH). Scores can be condensed into a physical component scale (PCS) and mental component scale (MCS). Rules for scoring can be found elsewhere [39]. 

### 2.4. Statistical Analysis

Data are presented as mean and standard deviation (SD), unless otherwise stated. The Kolmogorov–Smirnov test was performed to verify the normal distribution of the variables. One Sample *t*-test was performed to compare PA levels, %ST, QoL scores, Sum6-SK, absolute peak oxygen consumption (VO_2_peak), percentage of oxygen consumption (%VO_2_) at VT_1_, % VO_2_ at VT_2_, TUG-3m, and TUDS to reference values. Associations between PA and QoL variables were examined by Pearson’s and Spearman correlation analyses, depending on normal or non-normal distribution. The strength of the associations was classified as r_S_ ≤ 0.1 (very small), 0.1–0.29 (small), 0.3–0.49 (moderate), 0.5–0.69 (high), 0.7–0.89 (very high), and 0.9–1 (perfect). Binary logistic regressions were calculated to predict PCS and MCS based on anthropometric and strength variables after checking for multicollinearity. The level of significance was set at *p* < 0.05. The Statistical Package for the Social Sciences (SPSS) version 25.0 (SPSS Inc., Chicago, IL, USA) was used to perform all the statistical analyses.

## 3. Results

Clinical characteristics of the participants are shown at Table 1. Tanner Stages ranged II-IV: 7 (11.1%) II, 29 (46%) III, and 27 (42.9%) IV. A total of 50 participants were ≤15 years old. Dietary intake was set between 1800 to 2500 kcals/day with an average of 2350 ± 211.1 kcals/day.

### 3.1. Physical Activity

A summary of PA levels is shown at Table 2. Accelerometers were placed in the morning between 8:00–9:00 a.m. during weekdays and between 9:00–10:00 a.m. during weekends. Minimum and maximum wear times were 9.5 and 14.5 h, respectively. Weekdays and weekend days selected for the analysis corresponded to the same week. 

When comparing to the recommendations (M = 60 min/day) [22], total MVPA was significantly different (t [62] = −5.91 *p* < 0.001). A total of 22.2% of the AN patients met the MVPA criteria for children and adolescents. The male patients’ sample size did not allow for appropriate statistical power for comparisons (see Supplementary Table S1 for average PA levels broken down by sex). However, when analyzing cut-off values for a healthy PFit in females (*n* = 60), several significant differences were found. Relative ST (%) resulted significantly different (M_%ST_ = 76.8, SD = 10.2, t [59] = 5.125, *p* < 0.001) from cut-off threshold (M = 69%), with an ~82% of female patients showing a relative ST greater than the recommended. LPA and MPA values were significantly lower (M_LPA_ = 115.25 min/day, SD = 57.88; t [59] = −4.918, *p* < 0.001, and M_MPA_ = 24.77 min/day, SD = 13.46, t [59] = −5.308, *p* ≤ 0.001, respectively) from the recommended values for females (M_LPA_ ≥ 152 min/day, and M_MPA_ ≥ 34 min/day, respectively). Females not meeting the recommendations for LPA and MPA were 80% and 75%, respectively.

### 3.2. Physical Fitness

A summary of statistics broken down by PFit components is shown in Table 3. The percentage of oxygen consumption (%VO_2_) at VT_1_ resulted different (t [62] = −5.84) from the recommendations (M = 60%), with a total of 20.6% of the patients meeting the criteria for VT_1_. A total of 63.5% of the participants were able to reach VT_2_ (*n* = 41). Values of %VO_2_ at VT_2_ were significantly different (t [40] = 2.44) from recommendations (M = 80%), with 17.5% of the patients (*n* = 11) meeting the criteria. Absolute VO_2_peak and functional mobility (TUDS) values were significantly different (t [62] = −7.24; and t [62] = −4.88, respectively) compared to reference values (M_VO2peak_ = 2.1 L, and M_TUDS_ = 6.67 s) [40,41] with 84.1% and 77.8% of the AN patients not meeting the cut-offs for healthy values. Additional information on PFit variables broken down by sex are shown in Supplementary Table S1.

### 3.3. Body Composition

Body composition characteristics are presented in Table 4. Values of BMI resulted significantly different (t [62] = −11.04, *p* < 0.001) from the normative values (M = 18.5 kg/m^2^), with 44.4%of the patients meeting the criteria. Minimum %BF was met in the case of males (*n* = 3), and 60% (*n* = 36) of the females showed values below the norm (M = 21%). When analyzing the Sum-6SK, both females and males showed significantly lower values (t [59] = −10.55, *p* < 0.001; t [2] = −10.75, *p* = 0.009), compared to the reference values (M_Females_= 96 cm; M_Males_ = 68 cm) [42], with a total of 88.3% of females not meeting the cut-off. In addition, 28.3% of AN patients met the healthy values for arm circumference relaxed, while only 3.2% met the recommended values for CSA_ARM_ [43]. No reference values by sex and/or age were found for SMM, abdominal skinfold, mid-thigh CSA (CSA_THIGH_), and calf CSA (CSA_CALF_). Mean and SD on body composition variables broken down by sex are available on Supplementary Table S1.

### 3.4. Quality of Life

Physical and mental health related QoL is detailed in Table 5. Values of PF, RP, BP, VT, and RE resulted significantly different (t [62] = −5.98, t [62] = −10.14, t [62] = −8.75, t [63] = −13.37, and t [64] = −8.77, respectively) from the recommendations (M = 100) with 22.2%, 12.7%, 30.2%, 1.6%, and 23.8% of the patients meeting the criteria, respectively. Values of GH were different (t [62] = −4.22) from the recommendations (M = 75), and 33.3% of the patients met the criteria. SF scores resulted differently (t [62] = −6.88) from the recommendations (M = 90), and 22.2% met the criteria. MH values were different (t [62] = −3.90) from the recommendations (M = 70) with 38.1 % of the AN patients meeting the criteria. Lastly, the MCS values were different (t [62] = −5.22) from the recommendations (M = 50), with 30.2 % meeting the MCS criteria. No significant differences were found on PCS.

### 3.5. Association Analyses

Association analyses between QoL (SF-36 items and scales), PA and ST behaviors, and Pfit revealed small (0.1–0.29), moderate (0.3–0.49), and very high (0.7–0.89) significant associations (r_S_). Association matrix table is available as Supplementary Table S2.

Quality of life analyses revealed that PF scores were positively associated with 6RM-LR strength (r_S_ = 0.23, *p* = 0.04). GH scores correlated with 6RM-BP strength, arm circumferences, abdominal skinfold, Sum6-SK, and %BF (r_S_ = 0.23–0.60, *p* = 0.006–0.026). RE and MCS were positively associated with peak heart rate (r_S_ = 0.29–0.31, *p* = 0.013–0.023), and PCS with dietary intake and CSA_CALF_ (r_S_ = 0.26–0.29, *p* = 0.021–0.036). No associations were found between QoL and PA.

MVPA and vVPA were positively associated with relative VO_2_peak (r_S_ = 0.26–0.35, *p* = 0.005–0.037). Total PA was negatively associated with upper-thigh circumference (r_S_ = −0.27, *p* = 0.033), and VPA levels with BMI, abdominal skinfold, Sum6-SK, and %BF (r_S_ = −0.27–0.34, *p* = 0.005–0.032). Furthermore, vVPA correlated negatively with BMI, arm circumference, abdominal skinfold, Sum6-SK, and %BF (r_S_ = −0.25–0.33, *p* = 0.006–0.044). Sedentary behavior association analyses showed negative relationships between %ST, and MVPA, PA, LPA, and MPA (r_S_ = −0.46 to −0.95, *p* < 0.001).

Muscular fitness (6RM-LP, 6RM-BP and 6RM-LR) was positively associated with BW, BMI, and SMM (r_S_ = 0.23–0.53, *p* < 0.001–0.020). Circumferences (arm, upper, mid-thigh, and calf), skinfolds (abdominal skinfolds and Sum6-SK), CSAs (arm, thigh, and calf), and diastolic and systolic blood pressure were positively associated with upper and/or lower body muscular fitness. In addition, bench press and leg press strength (6RM-BP and 6RM-LP) was positively associated with absolute VO_2_peak, and peak ventilation (r_S_ = 0.29–0.48, *p* < 0.001–0.019).

Absolute VO_2_peak was positively associated with body composition variables [BW, BMI, anthropometric circumferences (arm, calf, upper- and mid-thigh), CSA (arm, thigh, and calf)]. Similarly, SMM was positively associated with percentage of VO_2_ at VT_1_, absolute VO_2_peak, peak ventilation, and agility (r_S_ = 0.23–0.42, *p* < 0.001–0.023). Additionally, upper and lower extremity CSAs were positively associated with peak ventilation, agility, and %VO_2_peak at VT_1_ (r_S_ = 0.29–0.49, *p* < 0.001–0.018).

### 3.6. Logistic Regression Analyses

Binary logistic regression was used to examine whether anthropometric and strength variables were associated with the likelihood of having normal PCS and MCS scores (see Supplementary Table S3). The predictor variable 6RM-LP was found to contribute significantly to the PCS-model [χ^2^ (1, *n* = 63) = 4.09, *p* = 0.043]. The model explained 9% (Nagelkerke R square) of the variance in PCS, and correctly classified a 70% of the cases. The estimated odds ratio (*OR*) indicated patients with greater 6RM-LP were 1.03 more likely of displaying healthy PCS scores. When evaluating MCS, the model was statistically significant [χ^2^ (2, *n* = 63) = 6.24, *p* = 0.044], suggesting that it could distinguish between those with and without healthy MCS scores. The model explained 13.4% (Nagelkerke R square) of the variance in MCS, and correctly classified 80% of the cases. Calf circumference contributed significantly, and the *OR* indicated that those patients increasing calf circumference were 0.73 less likely to have healthy MCS values.

## 4. Discussion

This is the first study to examine both PA levels after acute treatment using accelerometry and to evaluate the relationship of PA, PFit, sedentary behavior, and QoL. Overall, less than one quarter of the patients met the recommended MVPA [22], and poor QoL scores were associated with poor PFit, body composition and functional mobility. Further, excessive VPA and vVPA negatively impacted body composition (BMI, arm circumference, abdominal skinfold, Sum6-SK, and %BF), and aerobic and anaerobic function (%VO_2_ at VT_1_ and VT_2_, peak ventilation and absolute VO_2_ peak) was decreased and had a negative impact on BMI, circumferences, SMM and CSAs, and functional mobility. Therefore, PFit levels (i.e., cardiorespiratory fitness, body composition, muscular strength) and QoL scores remain poor even after proper weight restoration.

Previous research describing objective PA levels in an inpatient group of AN revealed that patients spent significant time in sedentary behaviors. Likewise, LPA levels were significantly increased during hospitalization (343.4 ± 151.9 min/day) [25], which contrasts the reduced LPA levels (113.1 ± 57.7 min/day) observed in our study. In addition, both groups of patients—under acute care [25] and after hospitalization—showed insufficient time spent participating in MVPA. Regarding the sex-specific cut-off thresholds to discriminate healthy versus unhealthy sedentary behaviors, our findings revealed significantly increased %ST compared to cut-off values (69% for females) [27], and similar ST values to patients under acute care [25]. When comparing the PA thresholds that discriminate between unhealthy and healthy CR fitness in female adolescents [27], AN patients did not reach the LPA, MPA, and MVPA values associated with a healthy CR fitness. Regular PA of different intensities is essential for the optimal development (physical and psychosocial) of all adolescents. Continuous engagement in PA and exercise of sufficient intensity (stimulus) has been shown to improve physical and mental health, quality of life, and prognosis in children and adolescents with different chronic conditions (i.e., cancer, cerebral palsy, cystic fibrosis, metabolic syndrome, etc.) [44,45,46,47]. Overall, our AN patients spent three quarters of their day participating in sedentary behaviors. Therefore, there is a potential benefit of engaging in more frequent time spent in LPA and MPA coupled with a decrease in ST. Our findings, together with those from Alberti and collaborators [25], suggest that each treatment stage may require from a different approach. More specifically, we theorize that potentiating LPA and MPA—through nutritionally supported and supervised exercise programs—and monitored time spent in vigorous-to-very vigorous activities could be most beneficial after acute treatment. However, patients under acute care may benefit from decreasing LPA [25] and implementing strategies to increase MPA.

In the present study, the aerobic and anaerobic function—examined through ventilatory thresholds (VT_1_ and VT_2_) relative to the peak capacity (% of VO_2_peak)—revealed that all patients reached VT_1_, however, three quarters were below the criteria which is indicative of a decreased aerobic function [48]. Regarding VT_2_, only 63.5% of participants reached the threshold, indicating that exercise at higher intensities (anaerobic function) is not sustainable and emphasizes patients’ low fitness level. Nevertheless, those patients able to reach VT_2_ (*n* = 11) showed values similar to normal healthy values (~80% of VO_2_max) [48]. Relative average values of CR capacity (rVO_2_peak) were similar to average values in healthy female adolescents [49]. However, rVO2peak (mL/kg/min) should not be considered in AN due to the overall misleading effect of low body weight and fat content. Instead, absolute VO_2_peak (L/min) will be more accurate in evaluating AN fitness level. Average absolute VO_2_peak reached in this study was lower compared with healthy adolescents (2.1–2.4 L/min) [40].

QoL assessment has been presented as an outcome variable in eating disorders [50], such that QoL worsens in patients with increased eating disorders and comorbid symptomatology [51]. Similarly, lower perceived QoL is associated with a more severe prognosis of AN [52]. In this cross-sectional study, we did not find significant associations between QoL scores and PA or sedentary behavior. However, the positive associations of QoL with muscular strength (i.e., lateral row and bench press 6RM) and body composition (i.e., arm circumferences, CSA_CALF_, summatory of skinfolds, and abdominal skinfold) suggest that poor muscular fitness and body composition are detrimental for AN patients’ physical and mental health after hospitalization. Supporting these findings, a meta-analysis examining CR and muscular fitness relationships with health-related QoL revealed that muscular fitness had a larger positive impact on physical and psychological health [53]. These results emphasize the potential of muscular fitness and QoL assessment to provide data on important aspects of physical and mental health in AN patients. The lack of association between PA and QoL, despite the positive association between muscular fitness and QoL, could be also linked to the complexity of objective quantification of loading activities. Activity monitors—accelerometers—have been validated to track mainly aerobic activity at different intensities (i.e., walking, running, etc.). As a result, the positive impact of loading activities on muscular fitness (i.e., muscle mass and strength) may go undetected, and with it their impact on QoL.

Research in PA, therapeutic exercise interventions, and QoL in AN is lacking. However, studies examining the effect of PA on QoL in patients with severe mental illness (i.e., schizophrenia, depression, etc.) show that PA is positively associated and a predictor of all QoL outcomes [54]. Moreover, Kane and colleagues reported that depression, poor emotional awareness, and low sense of control significantly predicted low QoL in AN [52]. Exercise has also shown to positively impact QoL in non-clinical adolescents [53]. Hence, QoL assessment in AN patients may be helpful in understanding the psychological factors (i.e., comorbid symptoms, emotional awareness) influenced by PA and exercise behaviors.

With the emerging evidence supporting therapeutic exercise engagement as a form of complementary treatment in eating disorders, care teams can begin to consider implementing structured physical exercise treatment planning. To ensure safe and effective PA management in AN, it is important to have a good understanding of the interrelations between PA, sedentary behaviors, PFit, and QoL at different stages of treatment. Standardized assessment of QoL, PA, sedentary behaviors, and PFit could help to improve our understanding of the relationships between these variables and disease prognosis. More specifically, our study suggests the need for the assessment of body composition beyond %BF, BW or BMI, and muscular fitness evaluation (i.e., upper and lower body 6RM) as key PFit factors impacting physical and mental health in AN after hospitalization.

This study has some limitations. There is a bias associated to the convenience sampling method that may limit the generalizability of our results to the greater of AN population. Nonetheless, the participants were recruited at a nationwide reference treatment unit. Another limitation is the participants in this study were mainly females. Although this corresponded to the expected proportion, it does not allow us to make inferences for adolescent male population. Future studies should include ample samples (i.e., larger size, males, uPA) to be able to generalize results. Further, examination of eating disorders and comorbid symptomatology in addition to the outcomes assessed in this study will increase the understanding of their impact in overall disease progress in addition to health related QoL.

Some important features strengthen this study. The experiment included adolescent participants that represent a very common age of onset in AN [55]. Another strength of the study was the sample was homogeneous (i.e., after acute treatment, receiving same treatment regime). Therefore, this result can be generalized to the group of female adolescents with AN in post-acute care under regular treatment. In addition, we did not only examine Pfit, but were also the first to measure PA and sedentary behaviors and their associations with QoL. Additionally, we established a relationship between decreased QoL and inadequate PFit after acute treatment, and exposed the need for further examination of the impact of sedentary behaviors and PA—including strength-related PA—in AN.

## 5. Conclusions

Anorexia nervosa patients after hospitalization treatment did not meet the daily PA criteria recommended, and compared to reference values, CR fitness, body composition and functional mobility remained deteriorated. However, QoL was positively associated with higher muscular fitness (i.e., strength, circumferences, CSAs). Likewise, muscular strength and absolute VO_2_peak were positively associated with body composition including SMM, CSAs, and circumferences. Therefore, management programs targeting improvements in PFit through increased muscular fitness, incorporating structured LPA and MPA, and reducing %ST, VPA and vVPA after hospitalization may be key for improving QoL and overall health closer to a healthy adolescent population.

## Figures and Tables

**Table 1:** Clinical characteristics of the sample.

	*n*	Mean		SD
Age (years)	63	13.5	±	1.15
BW_lowest_ (kg)	63	39.9	±	6.3
BMI_lowest_ (kg/m^2^)	63	15.8	±	1.6
Time to inclusion (days)	63	49.5	±	22.2
BW_gain_ (kg)	63	5.6	±	3.6

SD—standard deviation; BW_lowest_—lowest body weight; BMI_lowest_—lowest body mass index; BW_gain_—body weight regains until inclusion in the study.

**Table 2:** Physical Activity descriptors organized by weekdays, weekends, and average values.

	*n*	Total			Weekdays			Weekends		
		Mean		SD	Mean		SD	Mean		SD
LPA (min/day)	63	113.1	±	57.7	110.3	±	55.9	117.3	±	63.0
MPA (min/day)	63	24.3	±	13.4	24.0	±	14.8	24.8	±	16.5
VPA (min/day)	63	14.0	±	11.0	13.8	±	11.6	14.4	±	12.9
vVPA (min/day)	63	7.1	±	13.3	6.0	±	8.9	8.6	±	27.9
MVPA (min/day)	63	45.4	±	22.5	43.8	±	23.3	47.8	±	34.6
AT (min/day)	63	156.5	±	63.9	154.2	±	63.0	160.1	±	71.1
ST (min/day)	63	530.3	±	99.3	546.7	±	109.7	505.8	±	108.2
Relative ST (%)	63	77.0	±	10.1	77.7	±	10.0	75.7	±	11.2
Wear time (min/day)	63	686.9	±	78.4	700.9	±	91.0	665.9	±	89.9

SD—standard deviation; LPA—light physical activity; MPA—moderate physical activity; VPA—vigorous physical activity; vVPA—very vigorous physical activity; MVPA—moderate to vigorous physical activity; AT—active time; ST—sedentary time.

**Table 3:** Physical Fitness Levels.

	*n*	Mean		SD	Min	Max
Muscular Strength						
6RM-BP (kg)	63	43.5	±	7.3	31.8	65.8
6RM-LP (kg)	63	84.1	±	17.1	29.5	113.4
6RM-LR (kg)	63	43.1	±	8.7	25.0	63.5
Cardiorespiratory Fitness						
SBP (mmHg)	63	98.1	±	11.1	80.0	120.0
DBP (mmHg)	63	59.2	±	6.9	50.0	75.0
Time-end-test (min)	63	7.9	±	1.4	5.7	12.8
Speed-end-test (km/h)	63	7.1	±	0.7	5.1	9.3
Incline-end-test (%)	63	12.1	±	1.3	8.5	16.5
rVO_2_peak (mL/kg/min)	63	38.7	±	6.3	26.8	54.3
aVO_2_peak (L/min)	63	1.8	±	0.4 *	1.1	3.0
HRpeak (bpm)	63	187.4	±	9.4	167.0	213.0
VEpeak (L/min)	63	67.2	±	16.7	35.4	118.3
%VO_2_ at VT_1_	63	51.7	±	11.2 *	31.9	86.0
%VO_2_ at VT_2_	41	83.8	±	9.9 **	51.7	99.6
Functional Mobility						
TUG-3m (seconds)	63	4.2	±	0.3	3.5	4.9
TUG-10m (seconds)	63	9.5	±	0.8	7.9	11.1
TUDS (seconds)	63	6.2	±	0.7 *	4.8	9.7

SD—standard deviation; 6RM-BP—six maximum repetition bench press; 6RM-LP—six maximum repetition leg press; 6RM-LR—six maximum repetition lateral row; SBP—systolic blood pressure (mmHg); DBP—diastolic blood pressure (mmHg); aVO_2_peak—absolute peak oxygen consumption (L/min); rVO_2_peak—relative peak oxygen consumption (mL/kg/min); HRpeak—peak heart rate (bpm); VEpeak—peak ventilation (L/min); %VO_2_ at VT_1_—percentage of oxygen consumption at ventilatory threshold 1; %VO_2_ at VT_2_—percentage of oxygen of consumption at ventilatory threshold 2; TUG-3m—3 m timed-up-and-go; TUG-10m—10 m timed-up-and-go; TUDS— timed-up-and-down-stairs. * *p* < 0.001 compared to recommended values. ** *p* < 0.05 compared to recommended values.

**Table 4:** Body Composition Outcomes.

	*n*	Mean		SD	Min	Max
Height (m)	63	1.6	±	0.8	1.4	1.9
BW (kg)	63	45.4	±	7.5	29.3	71.6
BMI (kg/m^2^)	63	18.0	±	2.1	13.4	23.1
SMM (kg)	63	17.8	±	3.6	11.1	32.2
%BF	63	20.2	±	3.9	11.5	28.4
Sum6-SK (mm)	63	66.9	±	21.6	29.8	111.0
Abdominal-SK (mm)	63	14.2	±	5.5	2.4	25.5
Arm-C relaxed (cm)	63	22.9	±	2.4	17.0	29.7
Arm-C contracted (cm)	63	23.7	±	2.0	18.2	27.7
Upper-Thigh-C (cm)	63	49.4	±	5.0	32.7	59.0
Mid-Thigh-C (cm)	63	47.2	±	5.0	32.7	55.0
Calf-C relaxed (cm)	63	32.3	±	2.8	25.5	37.7
Calf-C contracted (cm)	63	32.8	±	3.0	25.5	38.8
Arm CSA (cm^2^)	63	23.1	±	6.3	11.5	51.3
Mid-Thigh CSA (cm^2^)	63	136.3	±	27.2	53.2	208.4
Calf CSA (cm^2^)	63	58.6	±	11.6	24.8	87.2

BW—body weight (kg); BMI—body mass index (kg/m^2^); SMM—skeletal muscle mass (kg); %BF—percent body fat; Sum6-SK—sum of six skinfolds thicknesses (biceps, triceps, subscapular, suprailiac, mid-thigh and medial calf sites) in mm; SK—skinfold thickness; C—circumference; CSA—muscle cross-sectional area.

**Table 5:** Quality of life.

	*n*	Mean		SD	Min	Max
PF	63	83.6	±	21.7 *	0.0	100.0
RP	63	66.8	±	26.0 *	0.0	100.0
BP	63	74.4	±	23.2 *	10.0	100.0
GH	63	64.9	±	19.0 *	18.8	100.0
VT	63	62.4	±	22.3 *	0.0	100.0
SF	63	65.5	±	28.3 *	12.5	100.0
RE	63	71.6	±	25.7 *	0.0	100.0
MH	63	57.9	±	24.7 *	0.0	100.0
PCS	63	51.3	±	7.9	28.8	64.6
MCS	63	41.1	±	13.5 *	6.2	67.4

PF—Physical functioning; RP—Role limitations due to physical health; BP—Bodily Pain; GH—General Health; VT—Vitality; SF—Social Functioning; RE—Role limitations due to emotional problems; MH—Mental Health; PCS—Physical Component Scale; MCS—Mental Component Scale. * *p* < 0.001 compared to recommended values.

## Data Availability

Due to ethical concerns, supporting data cannot be made openly available. The data that support the findings of this study are available upon request from the authors.

## Supplementary Materials

The following are available online at https://www.mdpi.com/article/10.3390/ijerph19052678/s1, Table S1: Physical Activity and Sedentary Behaviors, Muscular Strength, Cardiorespiratory Fitness and Body Composition broken down by sex, Table S2: Association Matrix of Quality of Life, Physical Fitness, Physical Activity and Sedentary Behavior Variables, Table S3: Logistic Regression Predicting the Likelihood of having of not healthy QoL scores.
