# Supplementary material for: Physical Fitness—Not Physical Activity Levels—Influence Quality of Life in Anorexia Nervosa
Source: Int J Environ Res Public Health. 2022 Feb 25;19(5):2678. doi: 10.3390/ijerph19052678 (PMC8910610; doi:10.3390/ijerph19052678)
Supplement: Supplementary file 1 [file ijerph-19-02678-s001.zip › ijerph-1573149/Supplementary Files/Supplementary Files Captions.pdf]

Supplementary Table S1. Physical Activity and Sedentary Behaviors, Muscular Strength, Cardiorespiratory Fitness, Body Composition and Quality of Life broken down by sex.

MVPA—moderate to vigorous physical activity; PA—physical activity; LPA—light physical activity; MPA—moderate physical activity; VPA—vigorous physical activity; vVPA—very vigorous physical activity; ST—sedentary time; 6RM-LP—six-repetition leg press; 6RM-LR—six-repetition maximum lateral raise; 6RM-BP—six-repetition maximum bench press; kcal—kilocalories; BW—body weight (kg); BMI—body mass index (kg/m^2); C—circumference; contr—contracted; SF—skinfold; Abd—abdominal; SK—skinfold; Sum6-SK—sum of six skinfolds; SMM—skeletal muscle mass (kg); %BF—body fat percentage; CSA—cross-sectional area; SBP—systolic blood pressure; DBP—diastolic blood pressure; t-end-test—time-end-test; v-end-test—speed-end-test; %-end-test — incline-end-test; aVO_2peak—absolute peak oxygen consumption (l/min); rVO_2peak—relative peak oxygen consumption (ml/kg/min); HRpeak—peak heart rate (bpm); VEpeak—peak ventilation (l/min); %VO_2 at VT_1—percentage of oxygen consumption at ventilatory threshold 1; %VO_2 at VT_2—percentage of oxygen of consumption at ventilatory threshold 2; TUG-3m—3 meters timed-up-and-go; TUG-10m—10 meters timed-up-and-go; TUDS— timed-up-and-down-stairs; PF—Physical functioning; RP—Role limitations due to physical health; BP--Bodily Pain; GH—General Health; VT—Vitality; SF—Social Functioning; RE—Role limitations due to emotional problems; MH—Mental Health; PCS—Physical Component Scale; MCS—Mental Component Scale.

Supplementary Table S2. Association Matrix of Quality of Life, Physical Fitness, Physical Activity and Sedentary Behavior Variables.

PF—Physical functioning; RP—Role limitations due to physical health; BP—Bodily Pain; GH—General Health; VT—Vitality; SF—Social Functioning; RE—Role limitations due to emotional problems; MH—Mental Health; PCS—Physical Component Scale; MCS—Mental Component Scale; MVPA—moderate to vigorous physical activity; PA—physical activity; LPA—light physical activity; MPA—moderate physical activity; VPA—vigorous physical activity; vVPA—very vigorous physical activity; ST—sedentary time; 6RM-LP—six-repetition leg press; 6RM-LR—six-repetition maximum lateral raise; 6RM-BP—six-repetition maximum bench press; kcal—kilocalories; BW—body weight (kg); BMI—body mass index (kg/m2); C—circumference; contr—contracted; SF—skinfold; Abd—abdominal; SK—skinfold; Sum6-SK—sum of six skinfolds; SMM—skeletal muscle mass (kg); %BF—body fat percentage; CSA—cross-sectional area; SBP—systolic blood pressure; DBP—diastolic blood pressure; t-end-test—time-end-test; v-end-test—speed-end-test; %-end-test — incline-end-test; aVO2peak—absolute peak oxygen consumption (l/min); rVO2peak—relative peak oxygen consumption (ml/kg/min); HRpeak—peak heart rate (bpm); VEpeak—peak ventilation (l/min); %VO2 at VT1—percentage of oxygen consumption at ventilatory threshold 1; %VO2 at VT2—percentage of oxygen of consumption at ventilatory threshold 2; TUG-3m—3 meters timed-up-and-go; TUG-10m—10 meters timed-up-and-go; TUDS— timed-up-and-down-stairs.

Supplementary Table S3. Logistic Regression Predicting the Likelihood of having of not healthy QoL scores

QoL - Quality of Life; MCS - mental component scales; C - circumference; 6RM-LP - six repetition maximum leg press; SE - standard error; df - degrees of freedom; Sig. - significance; OR - odds ratio; CI - confidence interval.
