# Supplementary material for: Physical Fitness—Not Physical Activity Levels—Influence Quality of Life in Anorexia Nervosa
Source: Int J Environ Res Public Health. 2022 Feb 25;19(5):2678. doi: 10.3390/ijerph19052678 (PMC8910610; doi:10.3390/ijerph19052678)
Supplement: Supplementary file 1 [file ijerph-19-02678-s001.zip › ijerph-1573149/Supplementary Files/Supplementary Table S1.pdf]

Supplementary Table S1. Physical Activity and Sedentary Behaviors, Muscular Strength, Cardiorespiratory Fitness, Body Composition and Quality of Life broken down by sex.

	Males				***Females***			
	**n**	**Mean**		SD	n	Mean		SD
Physical Activity								
**LPA (min/day)**	3	70.9	±	39.2	60	115.2	±	57.9
**MPA (min/day)**	3	15.2	±	8.4	60	24.8	±	13.5
**VPA (min/day)**	3	19.9	±	9.2	60	13.8	±	11.1
**vVPA (min/day)**	3	12.5	±	10.0	60	6.8	±	13.5
**MVPA (min/day)**	3	47.6	±	25.1	60	45.3	±	22.6
**AT (min/day)**	3	118.5	±	58.7	60	158.4	±	64.0
**ST (min/day)**	3	511.8	±	53.9	60	531.3	±	101.2
**Relative ST (%)**	3	81.3	±	8.8	60	76.8	±	10.2
**Wear time (min/day)**	3	630.3	±	39.2	60	689.7	±	79.0
Muscular Strength								
**6RM-BP (kg)**	3	55.9	±	13.3	60	42.8	±	6.4
**6RM-LP (kg)**	3	101.3	±	2.6	60	83.2	±	17.0
**6RM-LR (kg)**	3	53.7	±	11.2	60	42.5	±	8.3
Cardiorespiratory Fitness								
**SBP (mmHg)**	3	110.0	±	10.0	60	97.5	±	10.8
**DBP (mmHg)**	3	65.0	±	5.0	60	58.9	±	6.8
**Time-end-test**	3	8.3	±	0.2	60	7.9	±	1.4
**Speed-end-test**	3	7.5	±	0.3	60	7.0	±	0.7
**Incline-end-test**	3	12.8	±	0.3	60	12.1	±	1.4
**rVO2peak (ml/kg/min)**	3	45.4	±	3.1	60	38.3	±	6.3
**aVO2peak (l/min)**	3	2.5	±	0.4	60	1.7	±	0.3
**HRpeak (bpm)**	3	182.7	±	6.4	60	187.6	±	9.5
**VEpeak (l/min)**	3	103.8	±	22.0	60	65.4	±	14.3
**%VO2 at VT1**	3	64.7	±	21.8	60	51.1	±	10.3
**%VO2 at VT2**	2	78.2	±	7.9	39	84.0	±	10.0
Functional Mobility								
**TUG-3m (seconds)**	3	4.1	±	0.5	60	4.2	±	0.3
**TUG-10m (seconds)**	3	9.0	±	0.7	60	9.6	±	0.8
**TUDS (seconds)**	3	6.3	±	0.2	60	6.2	±	0.7
Body Composition								
**Height (m)**	3	1.7	±	0.1	60	1.6	±	0.7
**BW (kg)**	3	58.8	±	11.3	60	44.8	±	6.8
**BMI (kg/m^2)**	3	19.7	±	0.7	60	17.9	±	2.1
**SMM (kg)**	3	26.4	±	5.1	60	17.4	±	3.0
**%BF**	3	15.9	±	1.4	60	20.4	±	3.9
**∑6-SK (mm)**	3	35.7	±	5.2	60	68.5	±	20.9
**Abdominal-SK (mm)**	3	14.5	±	2.7	60	14.2	±	5.6
**Arm-C relaxed (cm)**	3	25.5	±	3.7	60	22.7	±	2.3
**Arm-C contracted (cm)**	3	25.2	±	1.0	60	23.6	±	2.0
**Upper-Thigh-C (cm)**	3	50.0	±	2.8	60	49.4	±	5.0
**Mid-Thigh-C (cm)**	3	50.0	±	2.8	60	47.1	±	5.0
**Calf-C relaxed (cm)**	3	35.9	±	0.5	60	32.1	±	2.7
**Calf-C contracted (cm)**	3	36.1	±	0.7	60	32.6	±	3.0
**Arm CSA (cm^2)**	3	35.6	±	13.8	60	22.4	±	5.2
**Mid-Thigh CSA (cm^2)**	3	184.6	±	21.2	60	133.9	±	25.3
Quality of Life								
**PF**	3	95.0	±	0.0	60	83.1	±	22.1
**RP**	3	58.3	±	25.3	60	67.2	±	26.2
**BP**	3	94.7	±	9.2	60	73.4	±	23.3
**GH**	3	69.2	±	9.5	60	64.7	±	19.3
**VT**	3	68.8	±	16.5	60	62.1	±	22.6
**SF**	3	66.7	±	31.5	60	65.4	±	28.4
**RE**	3	61.1	±	37.6	60	72.1	±	25.3
**MH**	3	53.3	±	29.3	60	58.1	±	24.7
**PCS**	3	57.0	±	2.3	60	51.0	±	8.0

**MCS**	3	37.0	±	18.2	60	41.3	±	13.4

MVPA—moderate to vigorous physical activity; PA—physical activity; LPA—light physical activity; MPA—moderate physical activity; VPA—vigorous physical activity; vVPA—very vigorous physical activity; ST—sedentary time; 6RM-LP—six-repetition leg press; 6RM-LR—six-repetition maximum lateral raise; 6RM-BP—six-repetition maximum bench press; kcal—kilocalories; BW—body weight (kg); BMI—body mass index (kg/m^2); C—circumference; contr—contracted; SF—skinfold; Abd—abdominal; SK—skinfold; Sum6-SK—sum of six skinfolds; SMM—skeletal muscle mass (kg); %BF—body fat percentage; CSA—cross-sectional area; SBP—systolic blood pressure; DBP—diastolic blood pressure; t-end-test—time-end-test; v-end-test—speed-end-test; %-end-test — incline-end-test; aVO_2peak—absolute peak oxygen consumption (l/min); rVO_2peak—relative peak oxygen consumption (ml/kg/min); HRpeak—peak heart rate (bpm); VEpeak—peak ventilation (l/min); %VO_2 at VT_1—percentage of oxygen consumption at ventilatory threshold 1; %VO_2 at VT_2—percentage of oxygen of consumption at ventilatory threshold 2; TUG-3m—3 meters timed-up-and-go; TUG-10m—10 meters timed-up-and-go; TUDS— timed-up-and-down-stairs; PF—Physical functioning; RP—Role limitations due to physical health; BP--Bodily Pain; GH—General Health; VT—Vitality; SF—Social Functioning; RE—Role limitations due to emotional problems; MH—Mental Health; PCS—Physical Component Scale; MCS—Mental Component Scale.
